# Circular RNA in colorectal cancer

**DOI:** 10.1111/jcmm.16380

**Published:** 2021-03-09

**Authors:** Anthony Li, Wei Cen Wang, Vivian McAlister, Qinfeng Zhou, Xiufen Zheng

**Affiliations:** ^1^ Department of Pathology and Laboratory Medicine Western University London Canada; ^2^ School of Medicine Queen’s University Kingston Canada; ^3^ Department of Microbiology & Immunology Western University London Canada; ^4^ Department of Surgery Western University London Canada; ^5^ London Health Sciences Centre London Canada; ^6^ Department of Laboratory Medicine Zhangjiagang TCM Hospital Affiliated to Nanjing University of Chinese Medicine Suzhou China; ^7^ Department of Oncology Western University London Canada; ^8^ Lawson Health Research Institute London Canada

**Keywords:** biomarker, circRNA, colorectal cancer, drug resistance, therapeutic target

## Abstract

Circular RNA (circRNA) is a highly abundant type of single‐stranded non‐coding RNA. Novel research has discovered many roles of circRNA in colorectal cancer (CRC) including proliferation, metastasis and apoptosis. Furthermore, circRNAs also play a role in the development of drug resistance and have unique associations with tumour size, staging and overall survival in CRC that lend circRNAs the potential to serve as diagnostic and prognostic biomarkers. Among cancers worldwide, CRC ranks second in mortality and third in incidence. In order to have a better understanding of the influence of circRNA on CRC development and progression, this review summarizes the role of specific circRNAs in CRC and evaluates their potential value as therapeutic targets and biomarkers for CRC. We aim to provide insight in the development of therapy and clinical decision‐making.

## 1 INTRODUCTION

The incidence and mortality of cancer are increasing every year, and projections for 2020 in the United States alone suggest 1.8 million new cancer cases and 600,000 cancer deaths.^1^ Colorectal cancer (CRC) ranks second in mortality and third in incidence of cancers worldwide and has a higher burden of disease in regions with high socioeconomic development.^2^ With 19 million disability‐adjusted life‐years (DALYs) and over 18 million years of life lost (YLLs) in 2017 alone, CRC continues to have a significant impact on global public health.^3^ Westernized diets, sedentary lifestyle behaviour and obesity are common modifiable risk factors.^4^ As the world becomes more digitalized in addition to increasing obesity rates and globalization of Western diets, the incidence of CRC is expected to only rise. This emphasizes the necessity to have a better understanding of CRC development and progression, with recent research suggesting that circular RNA (circRNA) has a crucial role to play.

CircRNAs are a type of single‐stranded non‐coding RNA that are highly abundant in mammalian cells.^5^ The majority of circRNAs are endogenously produced through a process known as back‐splicing which can result in exon shuffling.^6^ Given their covalently closed loop structure, lack of free terminals, and resistance to digestion by exonucleases and RNase R, circRNAs are more stable and evolutionarily conserved than linear RNAs.^7^ Research in recent years has discovered functions of circRNAs ranging from being microRNA (miRNA) sponges and transcription regulators to having protein interactions and allowing for translation.^8^, ^9^, ^10^, ^11^

**FIGURE 1:**
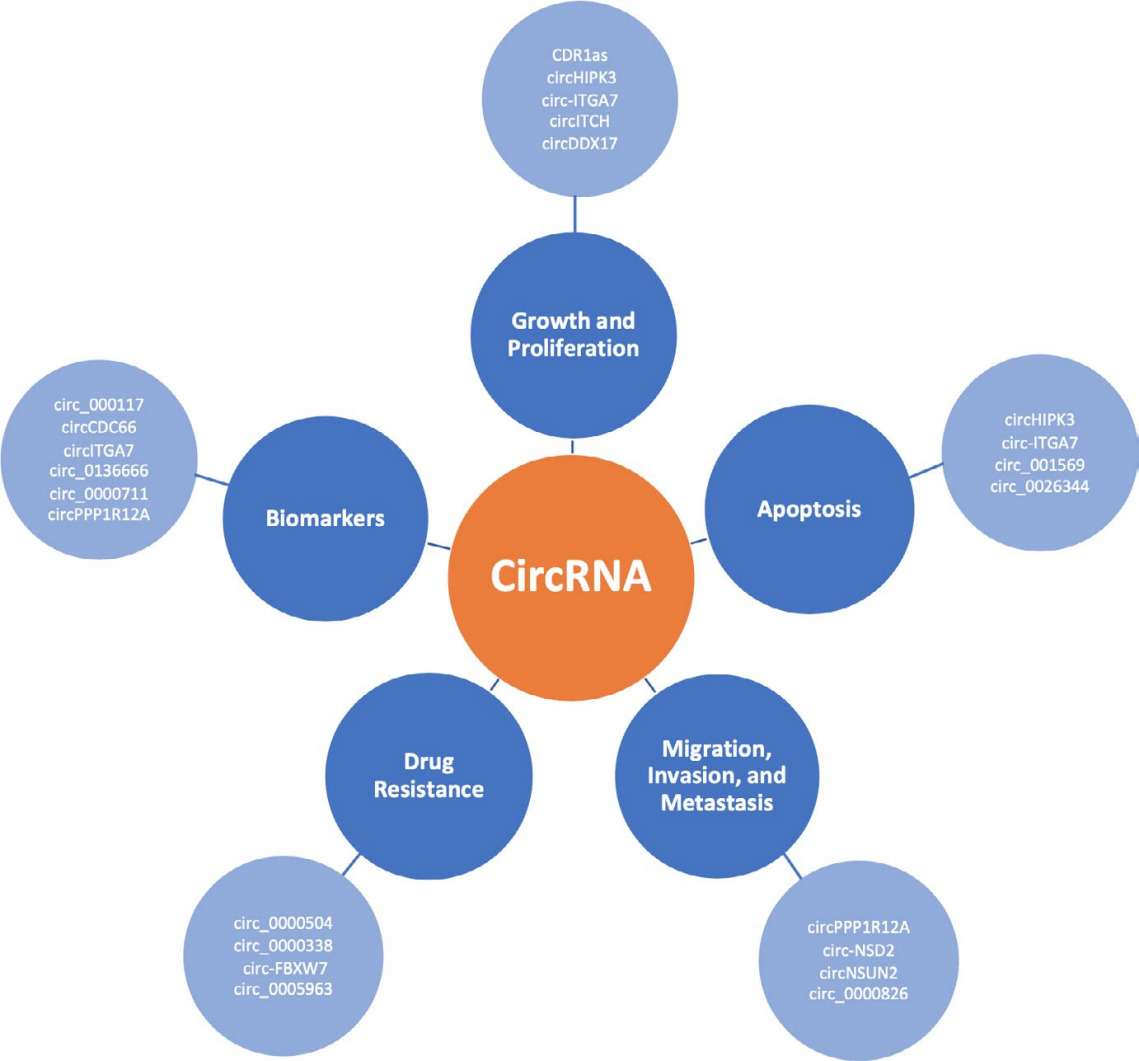
Roles of circRNA in colorectal cancer. CircRNAs have been identified to be involved in colorectal cancer growth, proliferation, apoptosis, migration, invasion, metastasis and drug resistance. Many circRNAs have also been proposed as potential diagnostic and prognostic biomarkers

Although the function and properties of many specific circRNAs remain unknown, novel research has eagerly explored the roles of circRNAs in human cancer (Figure 1). Some studies have discovered circRNAs with general oncogenic and tumour suppressor roles, with specific circRNAs changing gene expression in cancer cells.^12^, ^13^ Increasing research has shown that circRNAs are dysregulated in various stages of CRC and that circRNA levels could also be used as biomarkers for diagnosis and prognosis, even having the potential to serve as therapeutic targets. Certain circRNAs have been identified to be differentially expressed in drug‐resistant CRC cells.^14^ This suggests that circRNAs could serve as prognostic biomarkers and also aid in clinical decision‐making by focusing the treatment scope. This review aims to evaluate the potential value of circRNA in the diagnosis and treatment of CRC in clinical practice.

## 2 MECHANISTIC PRINCIPLES AND PROPERTIES OF CIRCRNA

### 2.1 Biogenesis of circRNA

The co‐transcriptional production of circRNAs could be mediated by three major mechanisms: intron‐pairing‐driven circularization, RNA‐binding protein (RBP)/trans‐factor‐driven circularization and lariat‐driven circularization.^15^

**FIGURE 2:**
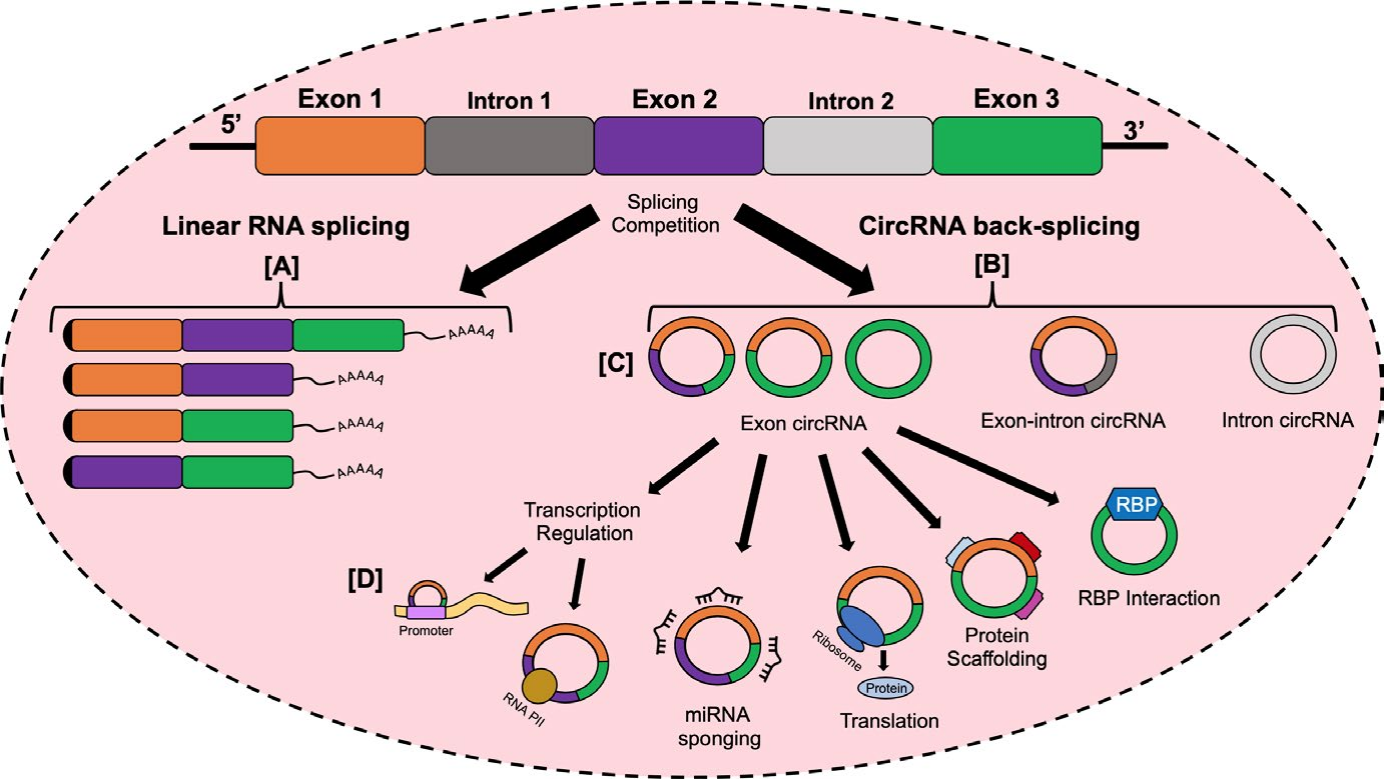
CircRNA biogenesis and interactions. A, The splicing of pre‐mRNA and removal of introns to form linear RNA. B, Back‐splicing of pre‐mRNA to form circRNA competing against linear RNA splicing. C, The three types of circRNA: Exon circRNA, exon‐intron circRNA and intron circRNA. D, Interactions of circRNA in transcription regulation through RNA polymerase II and promoter region binding, miRNA sponging, translation, protein scaffolding and RNA‐binding proteins (RBP)

CircRNAs are generally derived from the back‐splicing of RNA transcripts at canonical splice sites (Figure 2), which occurs in intron‐pairing‐driven and RBP/trans‐factor‐driven circularization.^5^, ^16^ Back‐splicing joins a downstream splice‐donor site with an upstream splice‐acceptor site. The region flanked by the splice‐donor and acceptor sites is then spliced out as a covalently closed circular form.^17^ The formation of a circRNA relies on the successful back‐splicing that competes against the splicing of pre‐mRNA.^17^, ^18^ In intron‐pairing‐driven circulation, the base pairing of long intronic sequences that contain inverted repeats, such as ALU elements, promote circularization.^19^ These events could result in exonic circRNAs (ecircRNA) or exon‐intron circRNAs (EIciRNAs) depending on the removal or retainment of introns in the circularized region.^20^ In RBP/trans‐factor‐driven circularization, RBPs (such as FUS and Quaking (HQK)) and trans‐factors dimerize and bind to specific motifs present in flanking introns to mediate circularization.^21^, ^22^ Furthermore, research in *Drosophila* demonstrated that circRNA biogenesis could also be influenced by the combinatorial actions of multiple heterogenous nuclear ribonucleoprotein and serine‐arginine proteins.^23^

In lariat‐driven circularization, lariats are first generated from exon skipping, where alternative exons are spliced out of the mRNA during canonical RNA slicing as part of the lariat. The lariat undergoes subsequent internal back‐splicing and intron removal to produce ecircRNAs.^5^, ^24^ Furthermore, intronic lariats containing a 7 nt GU‐rich element at the 5’ splice site and an 11 nt C‐rich element near the branchpoint can escape debranching and generate intronic circRNAs (ciRNAs).^25^

EcircRNA, ciRNA and EIciRNA constitute the three major classifications of circRNAs resulting from circRNA biogenesis (Figure 2C). The majority of circRNAs are ecircRNAs, which are located in the cytoplasm. In contrast, ciRNA and EIciRNA are mostly located in the nucleus.^20^

### 2.2 Detection of circRNA

The detection of circRNA can be challenging given that they have the same sequences as their linear RNA equivalents. Current techniques for the detection and quantification of circRNAs include circRNA microarray profiling, RNA‐sequencing (RNA‐seq) and reverse transcription‐quantitative polymerase chain reaction (RT‐qPCR) with divergent primers, northern blotting and in situ hybridization with probes specific to the back‐splice junction.^26^

### 2.3 General function of circRNA in cell activities

CircRNAs have been found to serve many functions, from acting as miRNA sponges and interacting with proteins to regulating transcription and allowing for translation. New functions and properties of circRNA continue to be discovered.

#### 2.3.1 CircRNA as miRNA sponges

One of the identified roles of circRNA is to function as miRNA sponges, with some circRNAs having multiple direct binding sites for miRNAs.^8^ Hence, the significance of those circRNAs in pathogenic pathways can be studied in the context of their target miRNAs. MiRNAs function to silence their target gene expression by partially base pairing with their home gene and negatively regulating gene expression.^27^ By sponging miRNAs, circRNAs are able to reverse the silencing effect of their target miRNA, thereby increasing the expression of the gene targeted by the miRNA.

#### 2.3.2 Regulation of transcription

Recent functional studies have found that circRNAs play a role in regulating the expression of RNA transcripts. Some circRNA can bind to RNA polymerase II, an enzyme for transcription, promoting and further regulating transcription.^15^ CircRNA can also affect their home gene expression through epigenetic regulation by binding to the promoter region.^28^ Given the co‐transcriptional biogenesis of circRNA, the expression of linear RNA often decreases when the majority of spliced transcripts circularises.^18^ CircRNAs can serve an important role in modulating gene expression given that they have been found to have differential expression relative to linear RNAs depending on their source tissue type.^29^, ^30^

#### 2.3.3 Translation of circRNA

The ability for circRNA to be translated has been heavily debated in the past given that circRNAs lack 5’ and 3’ terminal ends.^11^ However, increasing studies indicate the translation of circRNAs without a need for the 7‐Methylguanosine cap.^31^ The presence of a single N6‐methyladenosine site is sufficient for the initiation of translation, and through mass spectrometry, computational predictions and polysome profiling, a few endogenous circRNAs have been found to have the potential for translation.^32^

#### 2.3.4 CircRNA‐protein interactions

CircRNAs facilitate protein contact and assembly by acting as dynamic scaffolding molecules.^10^ Through these interactions, circRNA exert various functions. CircRNAs have been found to regulate transcription by mediating the recruitment of transcription factor complexes to promotor regions for transcription initiation.^33^ CircRNAs can also interact with transcription factors, serving as a protein scaffold for the formation of ternary complexes, thus increasing both overall expression and nuclear translocation.^34^ Moreover, circRNA‐protein interactions can suppress translation of proteins by competitively binding to mRNA transcripts, lowering protein translation rates.^35^

RBPs play a key role in post‐transcriptional gene expression by regulating the splicing, processing, localization and stabilization of circRNA.^36^ Some RBPs bind to RNA and promote back‐splicing, facilitating the production of circRNAs. Furthermore, there are circRNAs that are produced directly from RBP genes themselves, containing binding sites for their respective RBPs. Interestingly enough, since circRNAs are not translated in the same manner that linear mRNAs are, ribosomes do not displace any RBPs bound to circRNAs. As such, some RBPs can be bound to certain circRNAs with high specificity, significantly affecting biosynthesis, miRNA binding and expression levels.^18^

### 2.4 Properties of circRNA

#### 2.4.1 Abundance

CircRNAs are abundantly expressed across a diversity of eukaryotes, from humans and mice to zebrafish, *Drosophila*, and plants.^37^ Over one million circRNAs across six vertebrate species (human, macaque, mouse, rat, pig and chicken) are currently recorded in the circAtlas database.^38^

CircRNAs are widely expressed across numerous human cell types. There are 148,811 circRNAs originating from 12,251 genes in lung cells recorded in the circ2GO database as of 2020.^39^ In human fibroblast cells, 14.4% of expressed genes were found to produce circRNA and the expression of some circRNAs was 10‐fold higher compared to that of their associated linear transcripts.^5^ It was estimated that the total circRNA abundance was roughly 1% of the poly‐adenylated RNA abundance in A549, AG04450 and HeLa cells. CircRNA isoforms were more abundantly expressed than linear isoforms for around 50 genes in each cell line, while most genes expressed circRNA at 5‐10% of their linear isoforms.^40^ In leukocytes, circular isoforms were shown to constitute a substantial fraction of spliced transcripts belonging to hundreds of genes.^16^ Overall, the abundance of circRNAs provide a novel and valuable resource to investigate physiology and pathology of the body.

#### 2.4.2 Stability

One of the outstanding properties of circRNAs is their exceptional stability. CircRNAs are much more resistant to degradation by exonucleases as opposed to their associated linear mRNAs. RNase R is an exonuclease that requires a 3’ overhang of a minimum of seven nucleotides for tight binding and nuclease activity. Due to its circular nature, the absence of a 3’ overhang allows circRNAs to be preserved under RNase R treatment.^41^ Similarly, ribonuclease II and polynucleotide phosphorylase also preserve circRNA integrity while degrading their linear counterparts.^41^ CircRNAs demonstrated long half‐lives (over 48 hours) compared to their associated linear transcripts (less than 20 hours) when Hs68 cells were treated with actinomycin D.^5^

#### 2.4.3 Cell, tissue, developmental stage and disease‐specific expression

Multiple studies have demonstrated the cell‐type‐specific expression of circRNA.^40^, ^42^ CircRNAs are substantially up‐regulated in mouse brain tissue compared to heart, liver, lung and testis tissues. Explanations could be that the parent genes of many circRNAs are exclusively expressed in the brain or that the ratio between the relative circRNA transcript abundance and the total transcriptional output of the parent gene locus is higher in the brain compared to other tissues.^42^ CircRNA expression profiles were also different for distinct cardiac developmental stages in humans, demonstrating stage‐specific expression of circRNA.^43^ In mice, a global trend of circRNA up‐regulation was identified during the ageing process of neural tissues; however, no general trend was observed for linear RNA up‐regulation.^30^ The age‐dependent neural accumulation of circRNAs was also observed in *Drosophila*, where circRNA expression was far greater in adult heads compared to other larval, pupal and adult tissues.^44^ CircRNAs are found to be differentially expressed in diseases, such as diabetes mellitus, cardiovascular diseases, neurological disorders and cancer, compared to healthy controls.^45^, ^46^ The expression of many circRNAs is also correlated with the stages of cancer, such as in CRC.^47^, ^48^, ^49^

## 3 CircRNA AS THERAPEUTIC TARGETS IN CRC

### 3.1 Aberrant expression of circRNA in CRC

As described above, Alu elements regulate RNA transcript back‐splicing and circRNA biogenesis. The association of the mutation and methylation of Alu elements with CRC could be a possible explanation for aberrant circRNA expression.^50^, ^51^ Distinct expression profiles of circRNA between CRC and normal non‐malignant cells are thus anticipated and recent growing studies have supported this postulation. Moreover, the aberrant expressions of circRNAs are found to closely correlate with CRC pathology. The investigation of differentially expressed circRNAs between healthy and cancerous tissues and between primary and secondary tumours shines light on the novel role of circRNAs as potential CRC therapeutic targets.

#### 3.1.1 Tumour tissue versus healthy normal adjacent tissue

A global reduction of circRNA abundance was observed in CRC tissue and cell lines compared to normal colon tissue and global circRNA abundance was shown to be negatively correlated with proliferation. ^52^ Through microarray analysis, Zhang et al discovered 201 differentially expressed circRNAs out of 4,342 circRNAs detected between six pairs of CRC and normal colorectal tissue, of which 76 were up‐regulated and 125 were down‐regulated. Over 85% of differentially expressed circRNAs were located in exons.^53^ More recently, Ma et al reported the detection of 139 up‐regulated and 118 down‐regulated circRNAs in five CRC tissue samples compared to paired adjacent normal tissue through microarray analysis.^54^ Meanwhile, Li et al used RNA‐sequencing and detected 394 up‐regulated and 54 down‐regulated circRNAs in four CRC tissue samples compared to paired adjacent normal tissue.^47^ The discrepancy in the number of differentially expressed circRNAs between these studies could have been attributed to differences in the number of samples, method of circRNA detection and statistical analysis. Furthermore, the data were based on a limited number of circRNA junction reads, which could have impacted accuracy. Nonetheless, several circRNAs have been found to be consistently differentially expressed between CRC and adjacent healthy tissues. These include up‐regulated circRNAs such as ciRS‐7,^48^, ^55^, ^56^ circ‐HIPK3^56^, ^57^ and down‐regulated circRNAs such as circ‐ITGA7.^56^, ^58^, ^59^ Overall, a significant number of circRNAs are found to be differentially expressed between CRC and normal tissue, and their specific expression patterns may point to their potential involvement in CRC pathways.

#### 3.1.2 Primary tumour versus metastatic tissues

Metastasis is a major concern in CRC with approximately 20% of CRC patients already having metastasis at diagnosis.^60^ Lymph nodes are often the first metastatic site for CRC and other solid tumours. The liver is a common site for distant metastasis and is a leading cause of CRC mortality.^61^, ^62^ There have been 92 up‐regulated and 21 down‐regulated circRNAs identified in metastatic CRC tissue compared to primary CRC tissue.^63^ Differentially expressed circRNAs originated from all chromosomes except for the Y chromosome. The up‐regulated circ_0001178 and circ_0000826 in CRC metastasis were predicted to bind to multiple miRNAs and demonstrated high diagnostic potential for CRC metastasis.^63^ In a meta‐analysis of circRNA expression profiles in 1,430 CRC patients, differentially expressed circRNA in CRC correlated with tumour diameter, differentiation, TNM stage, invasion, and lymph node and distant metastasis. In particular, circRNAs up‐regulated in CRC correlated with worse overall survival, whereas circRNAs down‐regulated in CRC correlated with prolonged overall survival.^64^ Additionally, the comparison between primary SW480 cells and metastatic SW620 cells revealed 623 differentially expressed circRNA, of which 275 were up‐regulated and 348 were down‐regulated in SW620 cells.^65^ Many circRNAs, such as those derived from the *GLI3* and *RAPGEF5* genes, were down‐regulated to undetectable levels in SW620 cells despite high expression in SW480 cells. Meanwhile, circRNAs derived from the organic cation transporter SLC22A3 locus were detected in SW620 cells but absent in SW480 cells.^65^

#### 3.1.3 Tumour cell lines versus non‐malignant cells

Comparing the circRNA expression profile of the normal colon cell line NCM640 and the CRC cell lines SW480 and SW620, NCM460 contained a significantly higher number of circRNAs. Furthermore, 13,410 circRNAs were exclusively found in the NCM640 cell line, and 8,633 circRNAs were exclusively found in either SW480 or SW620 cells. There were 2,919 differentially expressed circRNAs between the NCM640 and CRC cell lines, of which 2,056 were between the NCM640 and SW480 cell lines and 1,758 were between the NCM640 and SW620 cell lines.^65^ Many studies confirmed differential expression of particular circRNAs between normal colon and CRC cell lines. Circ_000984 was highly expressed in CRC cell lines compared to normal colon cells and intestinal epithelial cells.^66^ Furthermore, circCCDC66 was detected in CRC cell lines Caco‐2, HCT116, HT‐29 and LS123, but absent in the normal colon‐derived cell line CCD 841 CoN.^67^ Yuan et al demonstrated that expression of tumour suppressor circ_0026344 was lower in CRC cell lines HCT116, SW480, SW620 and HT‐29, compared to normal colon cell lines FHC and NCM460.^68^ Li et al revealed that the expression of circ‐ITGA7 was down‐regulated in seven CRC cell lines, SW480, SW620, HCT116, RKO, Caco‐2, LoVo, and DLD1, compared to the normal colon cell line FHC.^58^

Distinguishing the expression profiles of circRNAs between normal versus CRC cells/tissue and between primary versus metastatic tumours lend circRNAs the potential to serve as therapeutic targets and diagnostic biomarkers.

### 3.2 Potential therapeutic targets for CRC

CircRNAs participate in a multitude of ways in CRC pathogenesis. Their functions are reflected in the consequences of their aberrant expressions in CRC. Understanding the role of circRNAs in CRC development and progression provides valuable insight into creating effective circRNA‐based therapeutic approaches. Potential therapeutic circRNAs, their mechanism of action and their parent genes are summarized in Table 1.

**TABLE 1:** CircRNA as therapeutic targets

CircRNA	Parent Gene	Expression in CRC	Target miRNA	Target Gene and Pathway	Role in CRC	Reference
CDR1as/CiRS‐7	CDR1	Up‐regulated	miR‐7	Up‐regulate EGFR, IGF1R, and RAF1	Promote: Proliferation; Growth; Invasion	48, 55
circHIPK3/circ_0000284	HIPK3	Up‐regulated	miR‐7	Up‐regulate FAK, YY1, IGF1R, and EGFR	Promote: Proliferation; Invasion; Migration Suppress: Apoptosis	^57^
circRNA‐ACAP2	ACAP2	Up‐regulated	miR‐21‐5p	Up‐regulate Tiam1	Promote: Proliferation; Migration; Invasion	^69^
circ_000984	CDK6	Up‐regulated	miR‐106b	Up‐regulate CDK6	Promote: Growth; Tumourigenesis	^66^
circ_0001955	CSNK1G1	Up‐regulated	miR‐145	Up‐regulate CDK6, MMP12, and RAB3IP	—	70, 106
circ_0055625	DUSP2	Up‐regulated	miR‐106b	Up‐regulate ITGB8	Promote: Proliferation; Migration; Invasion	49, 76
circ‐BANP	BANP	Up‐regulated	—	Down‐regulated phosphorylated Akt	Promote: Proliferation	^71^
circ_0000826	ANKRD12	Up‐regulated	—	—	Promote: Tumourigenesis; Migration; Invasion; Metastasis	63, 72
circPPP1R12A/ circ_0000423	PPP1R12A	Up‐regulated	—	Activate Hippo‐Yap pathway	Promote: Proliferation; Migration; Invasion	^73^
circ_0001178	USP25	Up‐regulated	miR‐382/587/616	Up‐regulate ZEB1	Promote: Migration; Invasion; Metastasis	63, 78
circ‐NSD2	NSD2	Up‐regulated	miR‐199b‐5p	Up‐regulate DDR1 and JAG1	Promote: Cell matrix interaction; Migration; Invasion; Metastasis	^79^
circ_001569	ABCC1	Up‐regulated	miRNA‐145	Up‐regulate E2F5, BAG4 and FMNL2	Promote: Proliferation; Invasion Suppress: Apoptosis	76, 80
circNSUN2	NSUN2	Up‐regulated	—	Stabilize HMGA2 transcript	Promote: Metastasis	^77^
circ‐ITGA7	ITGA7	Down‐regulated	miR‐370‐3p	Up‐regulate ASXL1, NF1, ITGA7 Down‐regulate RREB1 Suppress Ras signalling pathway	Promote: Apoptosis Suppress: Growth; Proliferation; Migration; Metastasis	58, 59
circDDX17	DDX17	Down‐regulated	—	—	Promote: Apoptosis Suppress: Proliferation; Migration; Invasion	^47^
circ_0026344	ACVRL1	Down‐regulated	miR‐21, miR‐31	—	Promote: Apoptosis Suppress: Proliferation; Invasion	68, 76
circ‐FBXW7	FBXW7	Down‐regulated	—	Activate PTEN Suppress NEK2 pathway and mTOR pathway	Suppress: Growth; Proliferation; Migration; Invasion	^82^
circITCH	ITCH	Down‐regulated	miR‐20a, miR‐7, and miR‐214	Up‐regulate ITCH Suppress Wnt/β‐catenin signaling pathway	Suppress: Proliferation	74, 75

#### 3.2.1 CircRNA in cell growth, proliferation and apoptosis of CRC

Many up‐regulated circRNAs in CRC have been demonstrated to promote cell growth and proliferation. Consequently, silencing their expressions effectively inhibits these processes and also promotes cell apoptosis. CDR1as, also known as CiRS‐7,^48^, ^55^, ^56^ and circHIPK3^56^, ^57^ are two of the top 10 most commonly found circRNAs, and both of them are up‐regulated in cancer. CDR1as promotes CRC progression by sponging the tumour suppressor miR‐7 and positively regulating the expression of miR‐7‐suppressed oncogenes such as EGFR, IGF1R and RAF1.^48^, ^55^ CDR1as knockdown decreased cell viability and colony‐formation capacity and increased the number of cells in the G_0_/G_1_ phase in CRC cells.^48^ CircHIPK3, also known as circ_0000284, is up‐regulated in CRC tissue and HT‐29, SW480 and SW620 cells. CircHIPK3 functions as an miR‐7 sponge and promotes CRC through positively regulating the expression of miR‐7 targets FAK, YY1, IGF1R and EGFR.^57^ Silencing circHIPK3 inhibited proliferation and induced apoptosis in HCT116 and HT‐29 cells.^57^ Altogether, this evidence strongly suggests that CDR1as and circHIPK3 potentially serve as significant therapeutic targets. Other circRNAs, such as circRNA‐ACAP2,^69^ circ_000984,^66^ circ_0001955^70^ and circ_0055625,^49^ have also been discovered as potential therapeutic targets for CRC and function through sponging miRNAs (Table 1). The knockdown of these circRNAs produced similar results of CRC growth and proliferation suppression while promoting apoptosis. These findings improve the understanding between the circRNA/miRNA regulatory networks and CRC pathogenesis and suggest the therapeutic potential of silencing these circRNAs or up‐regulating their target miRNAs. Moreover, the knockdown of circ‐BANP reduced CRC cell proliferation and the expression of phosphorylated Akt, suggesting that circ‐BANP could possibly promote CRC progression through the PI3K/Akt pathway.^71^ Hypoxia‐induced up‐regulation of circ_0000826 promoted CRC tumourigenesis in mouse xenograft models.^72^ In other cases, circRNAs contain ORFs and are capable of producing fully functional proteins. CircPPP1R12A, also known as circ_0000423, contains an ORF encoding the functional protein circPPP1R12A‐73aa which promoted the proliferation of CRC through activation of the Hippo‐Yap pathway.^73^

CircRNAs down‐regulated in CRC are often involved in the suppression of cell growth and proliferation, whereas their overexpression or ectopic expression enhance the inhibition of these processes and promote apoptosis. Similar to up‐regulated circRNAs, these down‐regulated circRNAs mostly function through sponging miRNAs. Circ‐ITGA7 is one of the top 10 most commonly found circRNAs and is also one of the most down‐regulated circRNAs in CRC.^56^, ^58^, ^59^ Ectopic expression of circ‐ITGA7 in SW480 and HCT116 cells suppressed proliferation, enhanced apoptosis and increased the number of cells in the G_1_ stage.^58^ Circ‐ITGA7 suppressed CRC proliferation by inhibiting the Ras signalling pathways through sponging miR‐370‐3p and up‐regulating the miRNA target, NF1.^58^ Circ‐ITGA7 could also sponge miR‐3187‐3p and up‐regulate the miRNA target, ASXL1.^59^ CircITCH expression is down‐regulated in CRC, and its overexpression decreased proliferation of HCT116 and SW480 cells.^74^ CircITCH negatively regulated the Wnt/β‐catenin signalling pathway through the up‐regulation of ITCH.^74^, ^75^ CircDDX17,^47^ circ_0026344^68^, ^76^ and circ‐FBXW7^77^ are also down‐regulated in CRC. The overexpression or ectopic expression of these three circRNAs also led to the suppression of CRC proliferation, suggesting their therapeutic potential. Overall, the current understanding of the involvement of these circRNAs in tumour pathways provides insight into their potential use to limit the growth and proliferation of CRC.

#### 3.2.2 CircRNA in migration, invasion and metastasis of CRC

CircRNAs up‐regulated in CRC usually promote the migration, invasion and metastasis of CRC. Silencing the expression of these circRNAs or up‐regulating the expression of their target miRNAs has been shown to reduce these processes. The previously mentioned CDR1as,^48^, ^55^ circHIPK3,^57^ circRNA‐ACAP2,^69^ circ_0055625,^49^ circPPP1R12A^73^ and circ_0000826^63^, ^72^ also promote CRC migration, invasion and metastasis, while their knockdown is effective in suppressing these processes. These circRNAs could potentially serve as promising therapeutic targets to limit not only growth and proliferation, but also the migration, invasion and metastasis of CRC. Circ_0001178 is up‐regulated in CRC and specifically promotes CRC migration, invasion and metastasis.^63^ Its knockdown inhibited cell migration and invasion in vitro and also inhibited lung and liver metastasis in vivo.^78^ Through sponging miR‐382/587/616, circ_0001178 up‐regulated ZEB1, a key initiator of epithelial to mesenchymal transition, and consequently promoted metastatic dissemination.^78^ Circ‐NSD2 is also up‐regulated in CRC liver metastatic tumours.^79^ It promoted cell matrix interaction and metastasis in HCT116 cells by sponging miR‐199b‐5p and up‐regulating the expression of miR‐199b‐5p targets DDR1 and JAG1. Silencing of circ‐NSD2 suppressed migration and invasion in HCT116 and RKO cells and decreased lung metastasis in mouse models, suggesting its potential as a therapeutic target for CRC metastasis.^79^ Circ_001569 is up‐regulated in CRC tissues and its knockdown inhibited proliferation and invasion, and promoted apoptosis in metastatic SW620 and LoVo cells.^80^ Circ_001569 could possibly promote CRC invasion through sponging miRNA‐145 and up‐regulating the miR‐145 targets E2F5, BAG4 and FMNL2, while also facilitating metastasis by activating the Wnt/β‐catenin signalling pathway.^80^, ^81^ Altogether, these results suggest that the inhibition of these up‐regulated circRNAs may reduce the migratory, invasive and metastatic potential of CRC. Additionally, circNSUN2, which was found up‐regulated in tissue and serum of CRC liver metastasis patients, formed a ternary complex with IGF2BP2 and HMGA2 RNA.^77^ This interaction enhanced the stability of the HMGA2 transcript and consequently promoted CRC liver metastasis. Silencing circNSUN2 in patient‐derived xenograft CRC cells inhibited tumour metastasis in liver and lung in mouse models. This demonstrated the potential for circRNA expression to be targeted directly, affecting CRC protein expression independent of miRNA.^77^

Down‐regulated circRNAs often have tumour suppressing roles in CRC migration, invasion and metastasis. Aside from suppressing CRC proliferation, circ‐ITGA7,^58^ circDDX17,^47^ circ_0026344^68^ and circ‐FBXW7^82^ function to inhibit tumour migration, invasion and metastasis. Consequently, their overexpression or ectopic expression reduced these processes both in vitro and in vivo. Taken together, these results suggest that up‐regulating the expression of these circRNAs could suppress CRC migration, invasion and metastasis, along with growth and proliferation.

Understanding the functions of circRNAs in CRC pathogenesis provides valuable insight into the potential for targeting circRNA in CRC therapeutics. Generally, the practicality of targeting up‐regulated circRNAs exceeds that of down‐regulated circRNAs due to their ease of detection and knockdown. Future therapeutic methods may also consider engineering exogenous circRNAs to target specific miRNAs or proteins to inhibit oncogenic pathways.

## 4 CircRNA AND DRUG RESISTANCE IN CRC

Chemoresistance of CRC has become increasingly challenging and prevalent, being a burden on effective treatment and associated with a poor prognosis.^83^ By looking at circRNA expression profiles of drug‐resistant CRC tissue and determining circRNAs mechanisms in tumour invasion and signalling, dysregulated circRNAs could serve as biotargets to increase chemosensitivity.

### 4.1 5‐fluorouracil resistance in CRC

Several circRNAs have been identified to confer drug resistance to CRC when dysregulated. 5‐fluorouracil (5‐FU), a pyrimidine analog, is the primary therapeutic component of regiments being offered to CRC patients.^84^, ^85^ However, with near 50% of CRC patients estimated to develop a level of resistance towards 5‐FU in late stages of treatment, the risk of mortality and the risk of recurrence both increase considerably due to limited remaining effective therapeutic options.^85^, ^86^ CircRNA expression profiles identified 71 circRNAs that were differentially expressed between 5‐FU resistant CRC cells and parental control cells.^86^ Three circRNAs in particular, circ_0007031, circ_0000504 and circ_0007006, were significantly up‐regulated. This dramatic modulation suggests their utility to predict the development of chemoresistance in CRC. Of these, circ_0007031 was up‐regulated 116‐fold in chemoresistant CRC cells and acted as a sponge for miR‐885‐3p, which is linked to the development of 5‐FU resistance.^87^ This overexpression of circ_0007031 has been suggested to play an important role in the development of 5‐FU resistance.^88^, ^89^ Thus, circ_0007031 has the potential to serve as both a therapeutic target to be down‐regulated and as a prognostic biomarker for treatment efficiency.

Circ_0000504 is highly up‐regulated in chemoresistant CRC cells.^86^ This circRNA sponges miR‐485‐5p, which negatively regulates STAT3, a transcription factor protein.^34^ The silencing of STAT3 has been found to significantly decrease the survival of 5‐FU resistant cells following treatment by 5‐FU and irradiation.^90^ Given that STAT3 is shown to increase chemosensitivity of 5‐FU resistant CRC cells, targeted down‐regulation of circ_0000504 would allow for the down‐regulation of STAT3, potentially allowing for 5‐FU resistance to be overcome.

### 4.2 Other drug resistances in CRC

Resistance to other drugs has been found to be conferred by various circRNAs as well. Several circRNAs have been identified to confer drug resistance to CRC when dysregulated. Microarray analysis of circRNA expression profiles identified 1,505 dysregulated circRNAs between chemosensitive and chemoresistant HCT116 CRC cells. Of these circRNAs, 773 were up‐regulated and 732 were down‐regulated, and the authors identified circ_32883 in particular as a biotarget for CRC drug resistance since it was found considerably up‐regulated in oxaliplatin‐resistant cells compared to chemosensitive CRC cells.^91^ Another study found that circ_0000338 increased FOLFOX drug sensitivity in CRC cells, while its knockdown resulted in increased chemoresistance.^91^ This suggests that circ_0000338 may increase the sensitivity of CRC cells to chemotherapy. However, when circ_0000338 was highly expressed in CRC exosomes, it was found to also have oncogenic properties, promoting viability against chemotherapy in chemoresistant cells compared to control.^92^ These different properties, given the oncogenic role in exosomes compared to the tumour suppressive role in CRC cells, suggest that circ_0000338 has a dual regulatory role, which is a feature that has been documented in certain miRNAs. Metastatic cancer cells can maximize their proliferation by expressing oncogenic miRNAs while exporting tumour suppressor miRNAs out of the cell.^93^ Thus, circ_0000338 may sponge exported tumour suppressor miRNAs when expressed in exosomes, highlighting the continued complexities and questions behind circRNA function.

Additionally, it was found that higher levels of circ_0005963 were detected in exosomes isolated from both oxaliplatin‐resistant patient serum and the oxaliplatin‐resistant cell line SW480/L‐OHP compared to levels in exosomes from oxalipatin‐sensitive samples.^88^ However, the oxalipatin‐sensitive cell line SW480 developed drug resistance to oxaliplatin both in vitro and in vivo when its levels of circ_0005963 were increased by treatment with circ_0005963 expression plasmids or by circ_0005963 enriched exosomes prepared from SW480/L‐OHP.^88^

By looking at circRNA expression profiles of drug‐resistant CRC tissue and determining circRNAs mechanisms in tumour invasion and signalling, dysregulated circRNAs could serve as biotargets to increased chemosensitivity.

## 5 CircRNA AS BIOMARKERS FOR CRC DEVELOPMENT

CircRNAs have several remarkable characteristics that provide tremendous potential for their use as biomarkers due to their abundance, stability, conservation across species, and disease‐specific and dynamic expressions. Table 2 provides a summary of the following circRNAs as potential biomarkers.

**TABLE 2:** CircRNA as biomarkers of CRC

CircRNA	Parent Gene	Expression in CRC	Type of biomarker	AUC Value	Reference
circ_0001178	USP25	Up‐regulated	Diagnostic marker	0.945	^63^
circCDC66	CCDC66	Up‐regulated	Diagnostic marker, Prognostic marker	0.884	^67^
circITGA7	ITGA7	Down‐regulated	Diagnostic marker	0.879	^58^
circ_0000567	SETD3	Down‐regulated	Diagnostic marker	0.865	76, 94
circ_0001649	SHPRH	Down‐regulated	Diagnostic marker	0.857	76, 95
circ_0003906	Chr6:29 989 443‐30 003 760 (no associated gene symbol)	Down‐regulated	Diagnostic marker	0.818	^96^
circ_0000826	ANKRD12	Up‐regulated	Diagnostic marker	0.816	^63^
circ_0000711	NFATC3	Down‐regulated	Diagnostic marker, Prognostic marker	0.810	76, 97
circ_001988	FBXW7	Down‐regulated	Diagnostic marker	0.788	76, 98
circPPP1R12A/circ_0000423	PPP1R12A	Up‐regulated	Diagnostic marker, Prognostic marker	—	^73^
CDR1as/CiRS‐7	CDR1	Up‐regulated	Diagnostic marker, Prognostic marker	—	48, 55
circCCDC66, circABCC1, circSTIL	CCDC66, ABCC1, STIL	Collectively Down‐regulated	Diagnostic marker	—	^100^
circNSUN2	NSUN2	Up‐regulated	Diagnostic marker	—	^77^
circ_0136666	PRKDC	Up‐regulated	Prognostic marker	—	76, 102
circ_0014717	CCNB1	Down‐regulated	Prognostic marker	—	76, 103
circ_100290	SLC30A7	Up‐regulated	Prognostic marker	—	76, 104
circ‐FBXW7	FBXW7	Down‐regulated	Prognostic marker	—	^82^
circ_0026344	ACVRL1	Down‐regulated	Prognostic marker	—	68, 76
circ_0122319, circ_0087391, circ_0079480, circ_0008039	PLOD2, AGTPBP1, ISPD, PRKAR1B	Up‐regulated	Prognostic marker	—	^105^

### 5.1 CircRNA as diagnostic markers

Receiver operating characteristic (ROC) curves are commonly used to assess the diagnostic ability of biomarkers. The area under the curve (AUC) of ROC is a value from 0 to 1 which summarizes the overall accuracy of the diagnostic test. A value of 0.5 demonstrates no discrimination between diseased and healthy states, while a value of 0.7‐0.8, 0.8‐0.9, and 0.9 and over suggests acceptable, excellent and outstanding diagnostic accuracy, respectively.

Circ_0001178 is up‐regulated in CRC and its AUC was 0.945, one of the highest values among all circRNAs, thus suggesting its outstanding diagnostic accuracy as a potential CRC diagnostic marker for CRC liver metastasis.^63^ CircRNAs with AUC between 0.8 and 0.9 include circCCDC66,^67^ circITGA7,^58^ circ_0000567,^94^ circ_0001649,^95^ circ_0003906,^96^ circ_0000826^63^ and circ_0000711,^97^ suggesting their excellent diagnostic accuracy as CRC biomarkers (Table 2). CircITGA7 was down‐regulated by 91.38% in the majority of CRC tissues compared to adjacent normal tissues.^58^ Circ_0000567 expression was negatively associated with tumour size and the staging of lymph, distal and tumour‐node metastasis.^94^ Circ_0001649 is down‐regulated in CRC, and its expression was negatively correlated with CRC pathological differentiation.^95^ Circ_0003906 is down‐regulated in CRC, and its low expression was correlated with lymph metastasis and poor differentiation.^96^ Finally, the AUC of the circ_001988 ROC curve was 0.788, suggesting acceptable accuracy as a diagnostic marker. Circ_001988 is down‐regulated in CRC, and its expression was shown to correlate with differentiation and invasion.^98^

The diagnostic potential of many other circRNAs have not been investigated using ROC curves, but their expressions are nonetheless closely related to the clinicopathological factors of CRC. It was found that the expression of circPPP1R12A was positively correlated with tumour proliferation, invasion and metastasis.^73^ CiRS‐7 is up‐regulated in CRC, and its expression was positively correlated with tumour size, stage and metastasis.^48^, ^55^ One study identified 623 differentially expressed circRNAs between the metastatic SW620 cells and the primary SW480 cells. The top 15 circRNAs were proposed as potential diagnostic markers for metastasis.^65^

CircRNAs are also abundant and highly stable in exosomes found in serum.^99^ RNA‐seq analysis demonstrated that circRNAs were enriched in secreted exosomes compared to the cells. Furthermore, circRNAs from cancer xenografts were shown to enter the circulation and were readily measured in the serum, suggesting the effectiveness and simplicity of diagnostic circRNA detection from the serum.^99^ Plasma levels of circ‐CCDC66, circ‐ABCC1 and circ‐STIL were significantly decreased in CRC compared to normal controls.^100^ The AUC of the ROC curve of the three‐circRNA panel was 0.78, which was higher than that of traditional protein biomarkers, carcinoembryonic antigen (CEA) and carbohydrate antigen 19‐9 (CA19‐9). The combination of the three‐circRNA panel with CEA and CA19‐9 demonstrated an AUC of 0.855, thus potentially improving diagnostic ability.^100^ Though its specific diagnostic accuracy has not been confirmed through ROC curve analysis, the expression of circNSUN2 was significantly increased in the serum from CRC with liver metastasis compared to primary CRC, suggesting its diagnostic potential.^77^ CircRNAs from serum have also been reported as potential diagnostic markers in other cancers, such as circ_0001785 for breast cancer.^101^

### 5.2 CircRNA as prognostic markers

CircRNAs can provide prognostic value, serving as markers for overall survival. Aside from their diagnostic potential, circCCDC66,^67^ circPPP1R12A,^73^ ciRS‐7^48^, ^55^ and circ_0000711 can also serve as prognostic markers,^97^ as suggested by the association between their respective aberrant expression in CRC and poor overall survival. Numerous studies also identified many other circRNAs with their respective aberrant expressions in CRC associated with poor overall survival, suggesting their prognostic potential. These include circ_0136666,^102^ circ_0014717,^103^ circ_100290,^104^ circ‐FBXW7^82^ and circ_0026344 (Table 2).^68^ The circScore based on a four‐circRNA panel of circ_0122319, circ_0087391, circ_0079480 and circ_0008039 developed by Ju et al can be used as a reliable prognostic tool for the recurrence of post‐operative diseases in stage II/III CRC patients. The inclusion of the circScore nomograms produced high accuracy in predicting the overall survival of stage II/III CRC patients.^105^

Increasing evidence has pointed towards the diagnostic and prognostic potentials of circRNAs for CRC. The use of circRNA alone or in combination with other conventional CRC biomarkers may provide more accurate screening for CRC patients, leading to earlier and more effective therapeutic intervention.

## 6 CONCLUSION

The potential applications offered by circRNAs continue to grow as we gain a better understanding of them. With the level of ease that circRNA can be collected from patients through saliva and blood, circRNA may one day be added to the arsenal of common clinical laboratory tests. Their ability to serve as biomarkers may even be used as screening tools for CRC prevention. The abilities to dampen miRNA function and to regulate protein function render circRNA not only as a master gene regulator in development and progression of CRC, but also as novel therapeutic targets with more robust effects for treating CRC. Results from in vitro cell culture models and preclinical animal models are providing us with a promising future where retuning circRNA expression in CRC patients will reduce CRC, DALY and YLL rates regardless of cancer stage, level of severity, or drug resistance. We recognize the complexity and numerous questions behind the many circRNAs that escape our understanding and hope that this is a priority for future research to elicit ground‐breaking change.

## CONFLICT OF INTEREST

The authors declare that they have no conflict of interests.

## AUTHOR CONTRIBUTIONS

**Anthony Li:** Conceptualization (equal); Data curation (equal); Visualization (equal); Writing‐original draft (equal); Writing‐review & editing (equal). **Wei Cen Wang:** Conceptualization (equal); Data curation (equal); Visualization (equal); Writing‐original draft (equal); Writing‐review & editing (equal). **Vivian McAlister:** Supervision (supporting); Writing‐review & editing (equal). **Qinfeng Zhou:** Writing‐review & editing (equal). **Xiufen Zheng:** Conceptualization; Funding acquisition (lead); Supervision (lead); Writing‐original draft (supporting); Writing‐review & editing (equal).

## Data Availability

Data sharing is not applicable to this article as no new data were created or analysed in this study.
